# Analogs of Natural 3-Deoxyanthocyanins: *O*-Glucosides of the 4′,7-Dihydroxyflavylium Ion and the Deep Influence of Glycosidation on Color

**DOI:** 10.3390/ijms17101751

**Published:** 2016-10-20

**Authors:** Nuno Basílio, Sheiraz Al Bittar, Nathalie Mora, Olivier Dangles, Fernando Pina

**Affiliations:** 1 LAQV-REQUIMTE, Department of Chemistry, Faculty of Science & Technology, Universidade NOVA de Lisboa, 2829-516 Monte de Caparica, Portugal; nuno.basilio@fct.unl.pt; 2 Faculty of Sciences, Technology & Health, University of Avignon, INRA, UMR408, 84000 Avignon, France; shirazbitar@yahoo.com (S.A.B.); nathalie.mora@univ-avignon.fr (N.M.)

**Keywords:** 3-deoxyanthocyanidin, flavylium, glucoside, photochromism, multistate

## Abstract

3-Deoxyanthocyanidins and their *O*-β-d-glucosides are natural pigments abundant in black sorghum. *O*-glycosidation can perturb the acid-base properties of the chromophore and lower its electron density with a large impact on the distribution of colored and colorless forms in aqueous solution. In this work, the influence of *O*-glycosidation on color is systematically studied from a series of 3-deoxyanthocyanin analogs. The pH- and light-dependent reversible reactions of 7-β-d-glucopyranosyloxy-4′-hydroxyflavylium (P3) and 4′-β-d-glucopyranosyloxy-7-hydroxyflavylium (P5) were completely characterized in mildly acidic solution and compared with the parent aglycone 4′,7-dihydroxyflavylium ion and the *O*-methylethers of P3 and P5. Except P5, the chalcone forms of the pigments exhibit a high *cis*-*trans* isomerization barrier that allows a pseudo-equilibrium involving all species except the *trans*-chalcone. At equilibrium, only the flavylium cation and *trans*-chalcone are observed. With all pigments, the colored flavylium ion can be generated by irradiation of the *trans*-chalcone (photochromism). Glycosidation of C7–OH accelerates hydration and strongly slows down *cis*-*trans* isomerization with the pH dependence of the apparent isomerization rate constant shifting from a bell-shaped curve to a sigmoid. The color of P5 is much more stable than that of its regioisomer P3 in near-neutral conditions.

## 1. Introduction

3-Deoxyanthocyanidins and their *O*-glycosides are natural pigments that are especially abundant in black sorghum [1] but also occur in other plants such as ferns [2,3,4]. 3-deoxyanthocyanidins of black sorghum are known to express a variety of health-promoting effects [5] and a better chemical stability [6] than the much more common anthocyanins, an important advantage for their potential development as natural food colorants.

**Figure 1:**
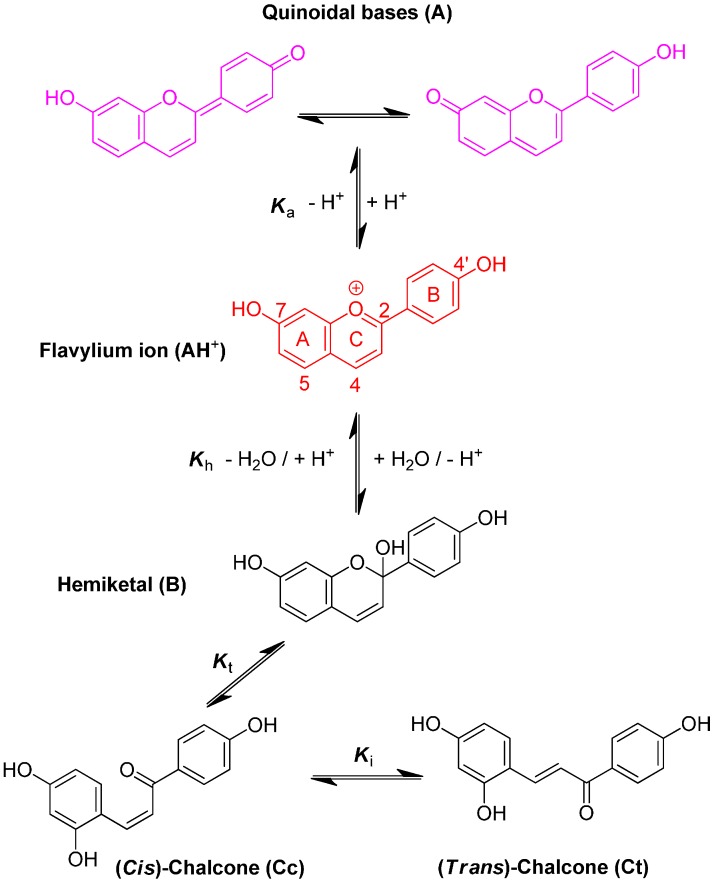
The reaction network of 4′,7-dihydroxyflavylium ion (DHF). With DHF, proton loss gives the 7-keto A tautomer (formation of the 4′-keto A tautomer requires glycosidation of C7–OH). The corresponding rate constants for the direct and reverse reactions are noted *k*_n_ and *k*_−n_, respectively (n = a, h, t, i). *K*_n_ = *k*_n_/*k*_−n_.

3-Deoxyanthocyanidins are typically isolated (sometimes, chemically synthesized), and thus represented, as the 4′,5,7-trihydroxyflavylium ion (apigeninidin) and its derivatives with additional OH and/or OMe groups (generally at C3′ and C5′). However, the flavylium ion (AH^+^) is stable only in highly acidic medium (pH < 2) and those pigments actually constitute a multistate system of chemical species, which are reversibly interconverted by external stimuli such as pH variations and light [7,8]. In the mildly acidic conditions typical of plant cell and food, the multistate system encompasses five distinct colored or colorless species (Figure 1). Understanding the flavylium multistate is of utmost importance for advanced (3-deoxy) anthocyanin research in plant, food and humans. As extraction of natural 3-deoxyanthocyanins is typically time-consuming and low-yielding, investigations on simpler analogs easily accessible by chemical synthesis may be a relevant alternative, especially for the systematic investigation of substituent effects. Moreover, synthetic flavylium ions are also an important class of photochromic compounds with several applications, such as models for optical memories or even for investigating basic properties of neurons [9].

**Figure 2:**
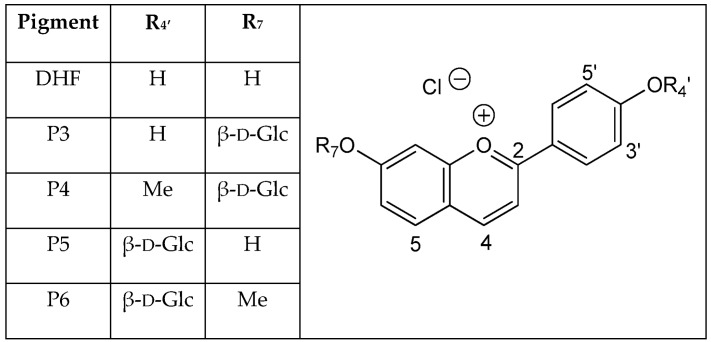
Pigments investigated in this work.

Hydroxyl groups at positions C4′ and C7 are common to all (3-deoxy)anthocyanins and critical to color development through the formation of the corresponding quinoidal bases (A). This is the purpose of this work to show that glycosidation of the 4′,7-dihydroxyflavylium ion (DHP, a simple apigeninidin analog lacking the OH group at C5), besides increasing its water solubility, has a deep and contrasted impact on its reaction network depending on the glycosidation site. Thus, the rate and thermodynamic constants pertaining to the reaction networks of the two *O*-β-d-glucosides P3 and P5 of 4′,7-dihydroxyflavylium (DHF) have been assessed. The *O*-methylethers of P3 and P5, respectively, noted P4 and P6, as well as DHF itself, are included for comparison (see structures in Figure 2).

A convenient way to characterize the flavylium-based multistate of chemical species is to carry out a direct pH jump by a fast addition of a small volume of strong base to equilibrated solutions. The first reaction that takes place upon a direct pH jump is the formation of quinoidal base A, Equation (1), by proton loss from the most acidic OH group, generally C7–OH. This reaction is fast and requires special equipment, such as temperature jumps or flash photolysis, to follow the corresponding kinetics [10,11]. The flavylium cation and the quinoidal base behave as a single species in the subsequent kinetic steps, which are by far much slower than proton transfer. Competing for the disappearance of the flavylium cation (and consequently the quinoidal base), water addition at C2 (hydration) gives hemiketal B, Equation (2). On the other hand, B is in equilibrium with *cis*-chalcone Cc with an interconverting rate falling in the sub-second timescale, i.e., sufficiently slow to be monitored by stopped-flow UV-visible spectroscopy, Equation (3).

AH^+^ + H_2_O ⇆ A + H_3_O^+^     *K*_a_ (acid-base) (1)

AH^+^ + 2H_2_O ⇆ B + H_3_O^+^     *K*_h_ (hydration) (2)

B ⇆ Cc         *K*_t_ (tautomerization) (3)

Cc ⇆ Ct          *K*_i_ (isomerization) (4)

Tautomerization is the second fastest process of the multistate, except in very acidic medium where hydration could be even faster. Finally, *cis*-*trans* isomerization leads to the *trans*-chalcone, Equation (4). This reaction could occur in less than one second up to days depending on the isomerization barrier. Brouillard and Dubois [10] discovered a peculiar characteristic of the (3-deoxy) anthocyanin multistate: the quinoidal base does not undergo water addition in acidic medium and the system evolves from Equation (1) to Equation (4) only by means of flavylium hydration. Thus, the quinoidal base can be regarded as a kinetic product of the flavylium reaction network.

With natural anthocyanins, i.e., 3-β-d-glycopyranosyloxyflavylium ions, isomerization is a minor process and the *trans*-chalcone typically represents less than 10% of the colorless forms [7]. Indeed, the trisubstituted double bond of anthocyanin chalcones has no marked preference for the *trans* vs. *cis* configuration. The situation is totally different with natural 3-deoxyanthocyanidins and the artificial analogs investigated in this work. Indeed, with such pigments, the *trans*-chalcone is essentially the sole colorless form at equilibrium in mildly acidic solution.

The flavylium multistate can be viewed as a single acid-base equilibrium involving the flavylium cation, on the one hand, and the overall conjugated base (CB), constituted by the mixture of quinoidal base, hemiketal and both chalcones, Equation (5):

(5) $$\begin{array}{c} {\mathrm{AH}}^{+}+H_{2}O⇆\mathrm{CB}+H_{3}O^{+}\;K\prime_{a}\\ \left[\mathrm{CB}]=[A]+[B]+[\mathrm{Cc}]+[\mathrm{Ct}\right] \end{array}$$

(6) $$K\prime_{a}=K_{a}+K_{h}+K_{h}K_{t}+K_{h}K_{t}K_{i}$$

## 2. Results and Discussion

### 2.1. Investigation of the 7-β-d-Glucopyranosyloxy-4′-hydroxyflavylium Ion (P3)

Natural (3-deoxy)anthocyanins and some related pigments such as P3 (see below) possess a high *cis*-*trans* isomerization barrier. This fact has a dramatic influence on the kinetics of the multistate. In particular, it is possible to define a pseudo-equilibrium, a long-life transient state where all species except the *trans*-chalcone are equilibrated. Analogous with the equilibrium, the pseudo-equilibrium can be defined by Equations (7) and (8):

(7) AH++H2O⇆CB^+H3O+          K^a[CB^]=[A]+[B]+[Cc]

*K*^_a_ = *K*_a_ + *K*_h_ + *K*_h_*K*_t_ (8)

Owing to the high *cis*-*trans* isomerization barrier, three distinct kinetic processes after a direct pH jump can be distinguished: (i) formation of the quinoidal base (not observed by stopped-flow); (ii) hydration followed by tautomerization, where the rate-limiting step is hydration; and (iii) *cis*-*trans* isomerization.

The three successive kinetic steps, all very well separated in time, are given by Equation (9) (too fast, not investigated in this work), Equations (10) and (11) [12]:

(9) $$k_{1\mathrm{st}}=k_{a}+k_{-a}[H^{+}]$$

(10) $$k_{2\mathrm{nd}}=\frac{\left[H^{+}\right]}{[H^{+}]+K_{a}}k_{h}+\frac{1}{1+K_{t}}k_{-h}[H^{+}]$$

(11) $$k_{3\mathrm{th}}=\frac{K_{h}K_{t}}{[H^{+}]+K_{a}+K_{h}+K_{h}K_{t}}k_{i}+k_{-i}$$

The alternative to direct pH jumps consists of adding a small volume of strong acid to equilibrated solutions of CB (at moderately acidic pH values) and is defined as reverse pH jumps. If the final pH is sufficiently low, dehydration becomes faster than tautomerization and four distinct steps can be observed: (i) conversion of the quinoidal base into flavylium cation during the mixing time of the stopped flow; (ii) dehydration of the hemiketal into flavylium cation, Equation (12); (iii) a slower step due to the conversion of the *cis*-chalcone into flavylium cation via the hemiketal, Equation (13); (iv) in a much slower process conversion of the *trans*-chalcone into flavylium cation, Equation (11). When the reverse pH jumps are carried out from pseudo-equilibrium, the kinetics steps (ii) and (iii) are better defined because the concentration of hemiketal and *cis*-chalcone is higher. This is especially important with the pigments investigated in this work as the *trans*-chalcone is practically the only CB component at full equilibrium. In Equation (13), the term *k*_t_ was not considered because dehydration is much faster and there is no reversibility. In other words, as soon as the hemiketal is formed from the *cis*-chalcone, it immediately gives the flavylium cation:

(12) $$k_{1\mathrm{st}\;\mathrm{reverse}}=\frac{\left[H^{+}\right]}{[H^{+}]+K_{a}}k_{h}+k_{-h}[H^{+}]$$

(13) $$k_{2\mathrm{nd}\;\mathrm{reverse}}=k_{-t}+k_{-t}^{H}[H^{+}]+k_{-t}^{\mathrm{OH}}[{\mathrm{OH}}^{-}]$$

The terms $k_{-t}^{H}$ and $k_{-t}^{\mathrm{OH}}$ account for the acid and base catalysis of *cis*-chalcone cyclization [13].

#### 2.1.1. pH Jumps

**Figure 3:**
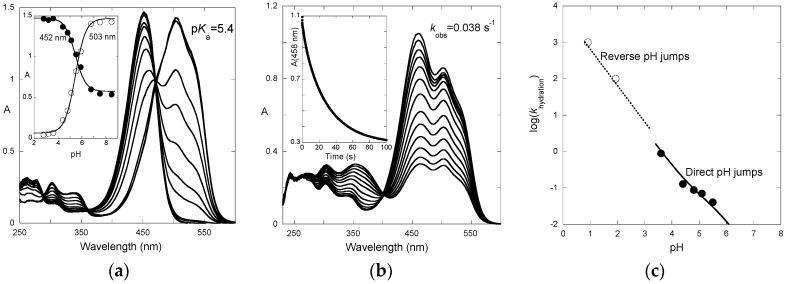
(**a**) pH-Dependence of the UV-visible spectrum of P3 taken 10 ms after a direct pH jump from pH 1.0 to higher pH values. On this timescale, the flavylium cation and the quinoidal base equilibrate with p*K*_a_ = 5.4; and (**b**) spectral variations upon a direct pH jump from pH 1.0 to 5.5 followed by stopped flow. Mono-exponential curve-fitting gives rate constant *k*_obs_ = 0.038 s^−1^; and (**c**) apparent rate constants of hydration for different pH values: (●) direct pH jumps, (◯) reverse pH jumps. Fitting was achieved for *k*_h_ = 0.08 s^−1^; *k-*_h_ = 9 × 10^3^ M^−1^·s^−1^; p*K*_a_ set at 5.4, *K*_t_ set at 0.6.

The absorption spectra of P3 after direct pH jumps (taken 10 ms after mixing, base formed during mixing time of solutions, ca. 6 ms) are shown in Figure 3a as a function of the final pH. The spectra are compatible with an acid-base equilibrium, Equation (1), with p*K*_a_ = 5.4. This value is consistent with the p*K*_a_ of 5.5 reported for 4′-hydroxyflavylium. However, it is much higher than the p*K*_a_ of 4.0 estimated for the corresponding aglycone (4′,7-dihydroxyflavylium), meaning that glycosidation at C7–OH cancels the most acidic OH group.

**Figure 4:**
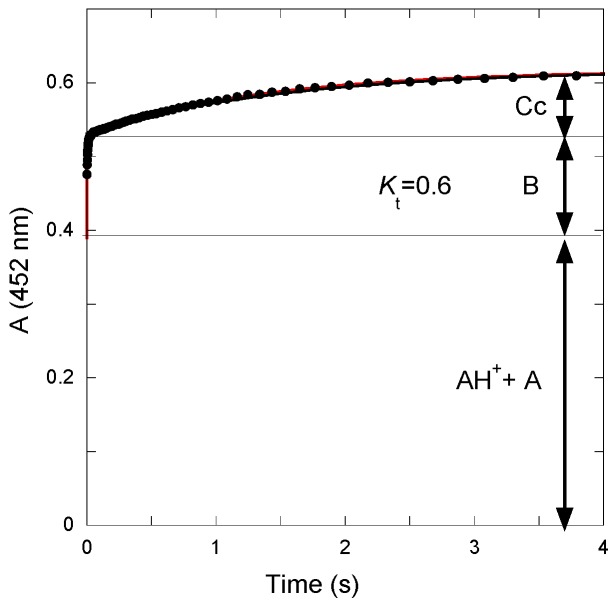
Reverse pH jump from a pseudo-equilibrated solution of P3 at pH = 4.65 (final pH = 1.9). Two kinetic steps are observed, the first one ascribed to dehydration, *k*_obs_ = 110 s^−1^, and the slowest to ring closure, *k*_−t_ = 0.6 s^−1^.

The *K*_t_ value was calculated from the amplitudes of the curves recorded after a reverse pH jump from pH = 4.65 to pH = 1.9 (Figure 4). The initial absorbance is due to the mixture of flavylium ion and quinoidal base present at pseudo-equilibrium at pH = 4.65. The first step corresponds to fast dehydration at pH = 1.9 (apparent rate constant = 110 s^−1^). The second step corresponds to the slower tautomerization. From the amplitudes of both steps, the *K*_t_ value can be estimated: *K*_t_ = 0.6.

The time-dependence of the UV-visible spectra after a direct pH jump is shown in Figure 3b for a final pH = 5.5. At this pH, the flavylium cation and quinoidal base are in near equal concentration. Both species vanish according to a first-order kinetics with apparent rate constant = 0.038 s^−1^ (half-life ≈ 18 s). The spectral variations are compatible with the formation of hemiketal and *cis*-chalcone at the expense of the colored forms.

The apparent rate constant for the first step of the reverse pH jumps from pseudo-equilibrium follows Equation (12) and was used to estimate *k*_−h_ as $k_{1\mathrm{st}}^{\mathrm{reverse}}\approx k_{-h}[H^{+}]$ for P3 at pH < 2 (Figure 3c). The apparent rate constant for the second step of the direct pH jumps follows Equation (10) and was used to estimate *k*_h_. One has: *k*_h_ = 0.08 s^−1^; *k*_−h_ = 9 × 10^3^ M^−1^·s^−1^. Thus, *K*_h_ = *k*_h_/*k*_−h_ = 8.9 × 10^−6^ M, p*K*_h_ = 5.05.

**Figure 5:**
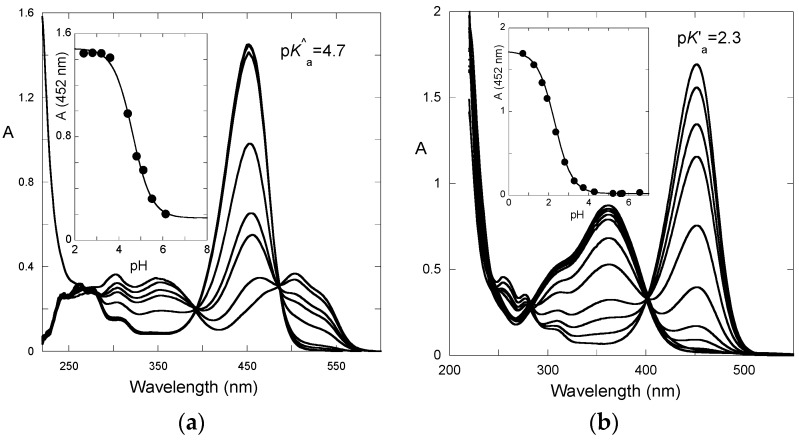
pH-Dependence of the UV-visible spectrum of P3; (**a**) recorded at pseudo-equilibrium (all species except the *trans*-chalcone); and (**b**) recorded at full equilibrium (all species including the *trans*-chalcone).

After a direct pH jump, a pseudo-equilibrium is established before formation of the *trans*-chalcone. The pH dependence of the UV-visible spectrum at pseudo-equilibrium can be fitted according to a single proton transfer with thermodynamic constant *K*^^^_a_ (Figure 5a). One obtains: p*K*^^^_a_ = 4.7.

**Figure 6:**
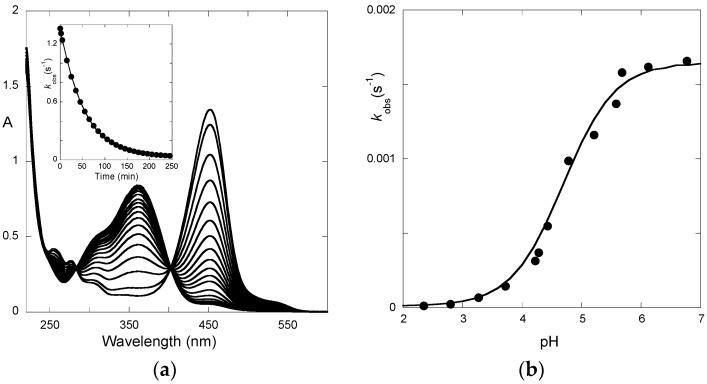
(**a**) spectral variations of P3 (5.4 × 10^−5^ M) after a direct pH jump from pH = 1.0 to pH = 3.3; *k*_obs_ = 3.1 × 10^−4^ s^−1^; and (**b**) plot of the apparent rate constant as a function of pH; curve-fitting was achieved with Equation (11): *K*_h_*K*_t_*k*_i_ = 3.4 × 10^−8^ M s^−1^; *k*_−i_ = 5.6 × 10^−6^ s^−1^; *K*^^^_a_ set at 10^−4.7^.

Finally, the spectral variations of P3 to reach full equilibrium from pseudo-equilibrium (third step of direct pH jumps, Figure 6) express the formation of the *trans*-chalcone at the expense of the other species. The pH-dependence of the corresponding first-order rate constant can be fitted with Equation (11) (*K*^^^_a_ set at 10^−4.7^), thus yielding: *K*_h_*K*_t_*k*_i_ = 2.9 × 10^−8^ M·s^−1^; *k*_−i_ = 5.9 × 10^−6^ s^−1^. Therefore, we deduce: *k*_i_ = 5.5 × 10^−3^ s^−1^, *K*_i_ = 935.

From the set of thermodynamic constants, the p*K*’_a_ value can be calculated to be 2.3, Equation (6), which is in perfect agreement with the value deduced from the pH-dependence of the UV-visible spectrum recorded on fully equilibrated solutions (Figure 5b).

#### 2.1.2. Photochemistry

In the case of flavylium multistates for which the *trans*-chalcone is the major species at equilibrium, it is generally possible to observe photochromism, i.e., color changes upon light absorption by this species [7]. Upon continuous irradiation, light absorption by the *trans*-chalcone of P3 leads to its disappearance with concomitant appearance of the flavylium cation (Figure S1). Then, the system reverts back thermally defining in this way a photochromic system.

**Figure 7:**
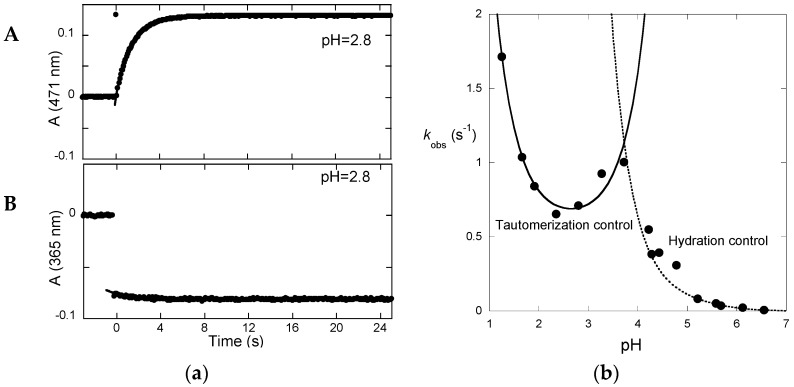
(**a**) Transient absorption upon a light flash applied to an equilibrated solution of P3 at pH = 2.8. A mono-exponential curve-fittings gives: *k*_obs_ = 0.7 s^−1^; **A**: flavylium formation; **B**: *trans*-chalcone consumption; and (**b**) plot of the apparent rate constant as a function of pH.

Irradiation of the *trans*-chalcone with a light pulse is another useful way to shift the multistate system from equilibrium (and follow the respective relaxation process) to obtain kinetic information (Figure 7a). Immediately after the flash, a fraction of *trans*-chalcone is converted into *cis*-chalcone in a few nanoseconds [14]. Because of the *cis*-*trans* isomerization barrier of P3, the recovery of the *trans*-chalcone is not immediately observed after the flash at pH = 2.8 and the system evolves only forward to the photochromic products, i.e., the flavylium cation. The dark reactions (after the flash) are thus tautomerization followed by dehydration. At pH = 2.8, hydration is faster than tautomerization and Equation (13) applies. In Figure 7a up, the raising of the visible absorption, featuring the Cc→AH^+^ conversion, is observed. At a wavelength where the chalcones absorb (Figure 7a down), the instantaneous bleaching after the flash is an indication of Ct→Cc conversion (Ct having a higher molar absorption coefficient than Cc), followed by a slower decrease consistent with the Cc→AH^+^ conversion (at 365 nm, Cc absorbs slightly more than AH^+^).

The pH-dependence of the apparent rate constant of the flash photolysis experiments (Figure 7b) clearly shows two distinct regimes [15]. At low pH, hydration is faster and the kinetics is controlled by tautomerization (Equation (13)). The shape of the branch is compatible with acid and base catalysis with *k_−_*_t_*^H^* and *k_−_*_t_*^OH^* respectively equal to 20 M^−1^·s^−1^ and 1 × 10^10^ M^−1^·s^−1^. At higher pH, hydration becomes slower and Equation (10) applies.

### 2.2. Investigation of the 4′-Glucopyranosyloxy-7-hydroxyflavylium ion (P5)

Unlike P3, P5 does not display a *cis*-*trans* isomerization barrier. With such pigments, after a direct pH jump, only two separated steps can be detected: (i) a very fast proton transfer to form the base; and (ii) a slow kinetic process yielding the *trans*-chalcone. Equation (14) can be deduced from the following assumptions [8]:

AH^+^ and A on the one hand, B and Cc on the other hand, are in fast equilibrium,
B and Cc taken collectively are in steady state,
  (14) $$k_{\mathrm{bell}}=\frac{\frac{\left[H^{+}\right]}{[H^{+}]+K_{a}}K_{h}K_{t}k_{i}+k_{-i}[H^{+}]}{[H^{+}]+k_{i}\frac{K_{t}}{k_{-h}}}$$

A plot of the apparent rate constant of Ct formation as a function of pH is a bell-shaped curve that tends to *k_−_*_i_ at low pH values (reverse pH jumps, rate-limiting isomerization) and to $k_{h}\frac{\left[H^{+}\right]}{[H^{+}]+K_{a}}$ at high pH values (direct pH jumps, rate-limiting hydration). At intermediate pH values (maximal rate of Ct formation), there is no rate-determining step and isomerization and hydration both contribute to the observed kinetics.

#### 2.2.1. pH Jumps

**Figure 8:**
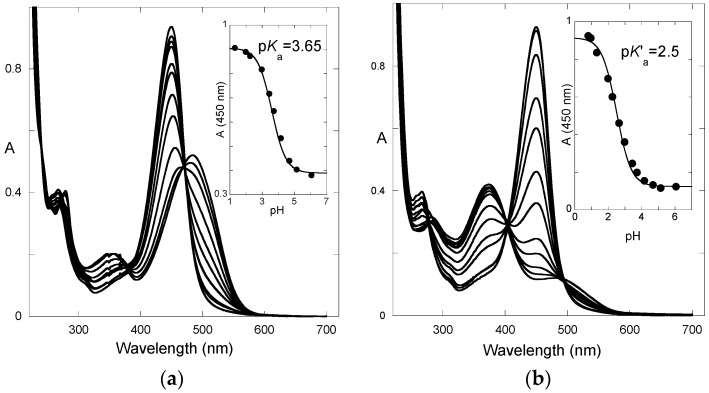
UV-visible spectra of P5 (4 × 10^−5^ M) recorded: (**a**) immediately after a direct pH jump; and (**b**) at equilibrium.

The pH-dependence of the UV-visible spectrum of P5 obtained 10 ms after direct pH jumps (Figure 8a), as well as at the equilibrium (Figure 8b), are compatible with the initial instantaneous formation of the quinoidal base and the final accumulation of the *trans*-chalcone.

**Figure 9:**
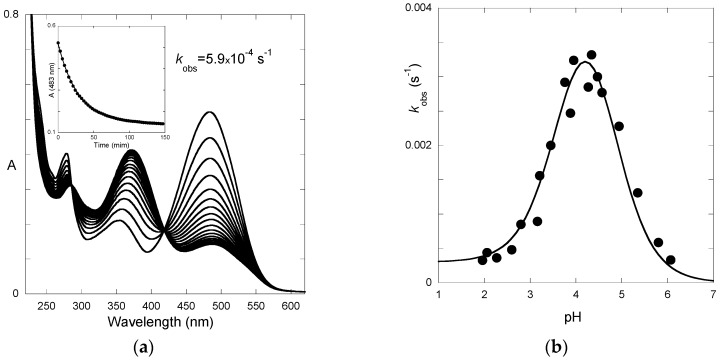
(**a**) time-dependence of the UV-visible spectrum of P5 after a direct pH jump from 1 to 5.8; and (**b**) pH-dependence of the apparent first-order rate constant of flavylium consumption and its fitting according to Equation (14): *K*_h_*K*_t_*k*_i_ = 8.5 × 10^−7^ M·s^−1^; *k*_−i_ = 3.0 × 10^−4^ s^−1^; *k*_i_*k*_t_/*k*_−h_ = 1.6 × 10^−5^ M·s^−1^ (*k*_h_ = 0.053 s^−1^), *K*_a_ set at 10^−3.7^.

The corresponding time-dependence of the spectrum is shown in Figure 9a for a direct pH jump to 5.8. Plotting the apparent first-order rate constant as a function of pH leads to the expected bell-shaped curve (Figure 9b), as observed for other flavylium ions bearing a hydroxyl group at C7.

#### 2.2.2. Photochemistry

With pigments having no *cis*-*trans* isomerization barrier, the rate of flavylium appearance after the flash is controlled by hydration, Equation (15), except at very low pH where hydration becomes faster than tautomerization, Equation (16). Control by hydration assumes B and Cc in equilibrium (taking into account a contribution of diffusion-controlled base catalysis of tautomerization). Equations (15) and (16) respectively correspond to Equations (10) and (13) used for P3 (slow isomerization) but include a term representing isomerization, which cannot be neglected for P5:

(15) $$k_{\mathrm{flash}\;(\mathrm{hydration})}=\frac{\left[H^{+}\right]}{[H^{+}]+K_{a}}k_{h}+\frac{1}{1+K_{t}}k_{-h}[H^{+}]+\frac{K_{t}}{1+K_{t}}k_{i}$$

(16) $$k_{\mathrm{flash}\;(\mathrm{tautomer}\;\mathrm{ization})}=k_{i}+k_{-t}+k_{-t}^{H}[H^{+}]+k_{-t}^{\mathrm{OH}}[{\mathrm{OH}}^{-}]$$

The flash photolysis experiments at pH = 4.2 are reported in Figure S2a. After the flash, Ct (λ_max_ = 360 nm) is instantaneously converted into Cc (lower molar absorption coefficient at this wavelength), and then rapidly regenerated due to the lack of *cis*-*trans* isomerization barrier, in competition with flavylium formation (λ_max_ = 450 nm).

The pH dependence of the corresponding apparent first-order rate constants shows the change from hydration regime (higher pH, Equation (15)) to tautomerization regime (lower pH, Equation (16)). The curve-fittings (Figure S2b) were achieved for *k*_h_ = 0.053 s^−1^, *k*_−h_/(1 + *K*_t_) = 9 × 10^3^ M^−1^·s^−1^; *K*_t_*k*_i_/(1 + *K*_t_) = 0.15 s^−1^, *k*_−t_ + *k*_i_ = 1.35 s^−1^; $k_{-t}^{H}$ = 25 M^−1^·s^−1^; $k_{-t}^{\mathrm{OH}}$ = 2 × 10^10^ M^−1^·s^−1^ (*K*_a_ set to 10^−3.7^) in good agreement with the data from the bell-shaped curve.

The quantum yield, i.e., the percentage of incident light energy used for the Ct→Cc conversion, is a useful parameter for a complete estimation of the rate and equilibrium constants [13]. Its apparent value φ can be estimated from the percentage of color generated after illumination. It thus reflects the pH-dependent contribution of the forward process (Cc→B→AH^+^ + A). When hydration is the rate-determining step (tautomerization equilibrium achieved), the apparent rate constants for the forward and backward reactions are, respectively, the first and the last parts of Equation (15). Neglecting the contribution of *k*_h_[H^+^]/([H^+^] + *K*_a_), the pH-dependence of the quantum yield is given by Equation (17):

(17) $$\Phi =\Phi_{0}\frac{k_{\mathrm{forward}}}{k_{\mathrm{forward}}+k_{\mathrm{backward}}}=\frac{\left[H^{+}\right]}{[H^{+}]+\frac{K_{t}k_{i}}{k_{-h}}}$$

Analogously, when the rate-determining step is tautomerization, the pH-dependence of the quantum yield is easily deduced from Equation (16) and follows Equation (18):

(18) $$\Phi =\Phi_{0}\frac{k_{-t}+k_{-t}^{H}{[H}^{+}]+k_{-t}^{\mathrm{OH}}{[\mathrm{OH}}^{-}]}{k_{-t}+k_{-t}^{H}{[H}^{+}]+k_{-t}^{\mathrm{OH}}{[\mathrm{OH}}^{-}]+k_{i}}$$

**Figure 10:**
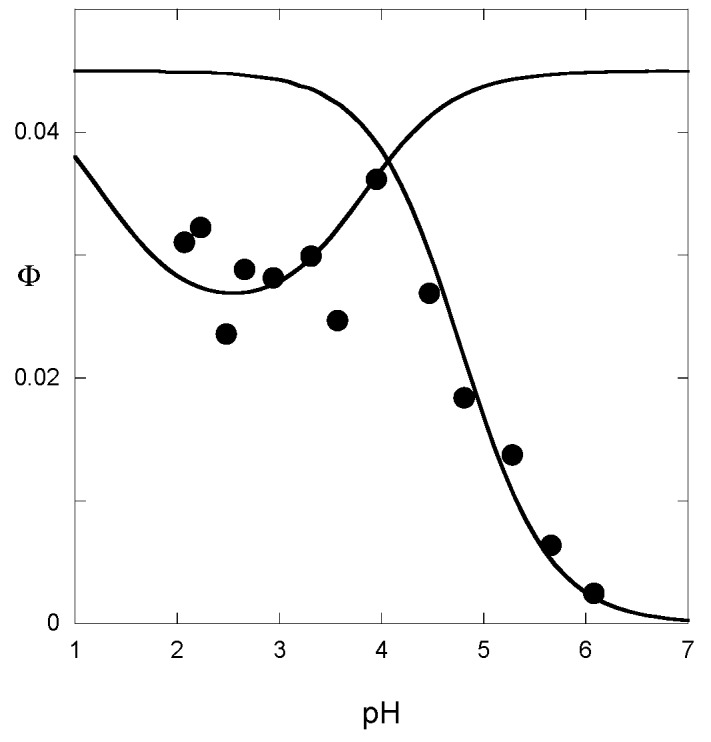
Quantum yield of P5 as a function of pH. Fitting was achieved with Equations (17) and (18) for *k*_i_*k*_t_/*k*_−h_ = 1.6 × 10^−5^ M s^−1^; *k*_i_*K*_t_/(1 + *K*_t_) = 0.15 s^−1^; *k*_−h_/(1 + *K*_t_) = 9 × 10^3^ M^−1^·s^−1^; $k_{-t}^{H}$ = 25 M^−1^·s^−1^; $k_{-t}^{\mathrm{OH}}$ = 2 × 10^10^ M^−1^·s^−1^.

From the curve-fitting of the φ vs. pH plots according to Equations (17) and (18) (Figure 10), the *k*_i_ value can be estimated, thereby allowing the calculation of the other constants.

### 2.3. Substituent Effects on the Flavylium Multistate

**Figure 11:**
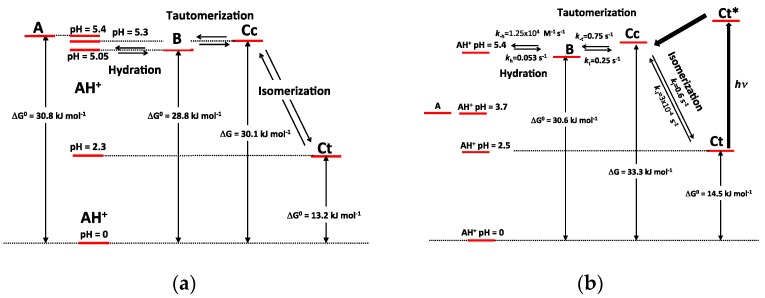
Energy level diagrams of P3 (**a**) and P5 (**b**). *: Excited state.

The investigations of P3 and P5 were reproduced with the corresponding *O*-methylethers P4 and P6 and with the parent 4′,7-dihydroxyflavylium ion (DHF) itself. Although DHF has already been studied [7], the complementary analysis of the quantum yield (deduced from the flash photolysis experiments) conducted in this work permitted a more accurate determination of *k*_t_ and *k*_−t_. The complete set of thermodynamic and kinetic constants characterizing the multistates of the five pigments is reported in Table 1. The energy level diagrams of P3 and P5 are also represented in Figure 11.

The major consequence of methylation is obviously to cancel the acid-base equilibrium (Equation (1)). While the influence of methylation on the hydration and tautomerization steps is marginal, its impact on the *cis*-*trans* chalcone isomerization is very significant. Interestingly, only DHF and P5, i.e., the two pigments with an OH group at C7, display a low isomerization barrier, which prevents the establishment of the pseudo-equilibrium (Equation (7)). Glycosidation or methylation of C7–OH (P3, P4, P6) raises the barrier and makes the *cis*-*trans* chalcone isomerization a slow kinetically distinct process, as observed with natural anthocyanins. However, the influence of glycosidation is much more pronounced. For instance, there is a ca. 100-fold difference between the *k*_i_ values of the two regioisomers P3 and P5, and glycosidation of C7–OH (P3 vs. DHP) lowers *k*_i_ by a factor ca. 50, vs. only 7.5 for methylation (P5 vs. P6). By contrast, glycosidation of C4′-OH (P5 vs. DHP) moderately accelerates isomerization (a factor of ca. 2).

**Figure 12:**
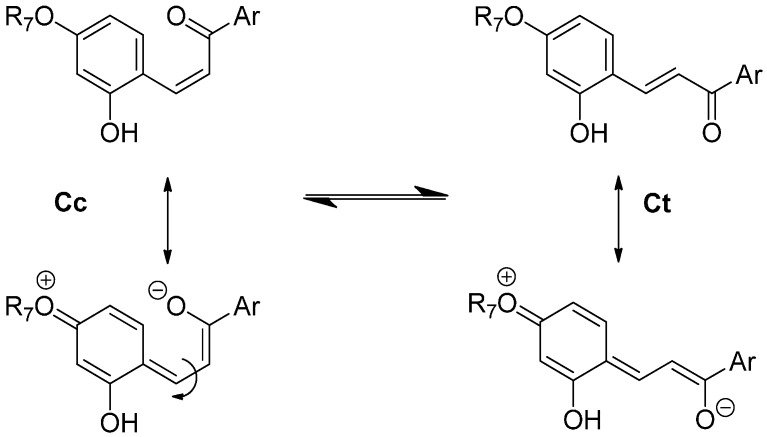
Electron donation from the substituent at C7 (R_7_ = H, Me, Glc) and its accelerating effect on *cis*-*trans* chalcone isomerization.

As a powerful electron-donating group at C7 is expected to weaken the C=C bond of chalcones through electron-delocalization (Figure 12), it may be concluded that the Glc moiety of P3 and P4 (through the combination of the electron-withdrawing effects of its *O*-atoms) largely quenches the electron-donating capacity of the *O*-atom at C7. Although much less pronounced, this effect is also significant with flavylium ions. Indeed, water addition to P3 and P4 is four times as fast as with DHP. It is thus likely that lowering the electron-donating capacity of the substituent at C7 increases the fraction of positive charge at C2 and consequently the hydration rate constant.

The apparent rate constant of color loss for P3 increases with pH to reach a plateau value at pH 6–7 (Figure 6b). This trend reflects the rate-limiting isomerization and the higher fraction of *cis*-chalcone when the pH increases. By contrast, above pH = 4, the apparent rate constant of color loss for P5 decreases with pH (Figure 9b). In the absence of a high *cis*-*trans* isomerization barrier, hydration becomes the rate-limiting step and the slower fading now reflects the lower fraction of flavylium ion when the pH increases. These subtle changes make the P5 color much more stable than the P3 color in mildly acidic to neutral conditions (at pH 6, the half-life of color is ca. 40 min for P5 vs. ca. 7 min for P3). By contrast, the coloring potential of P5 in such conditions is moderated by the quinoidal base (proton loss at C7–OH) having ca. half the molar absorption coefficient of the corresponding flavylium at λ_max_ (Figure 8a), while, for P3, both flavylium and quinoidal base (proton loss at C4′–OH) have roughly the same capacity for light absorption at λ_max_ (Figure 3a).

### 2.4. Substituent Effects on the Performance of the Photochromic System

**Figure 13:**
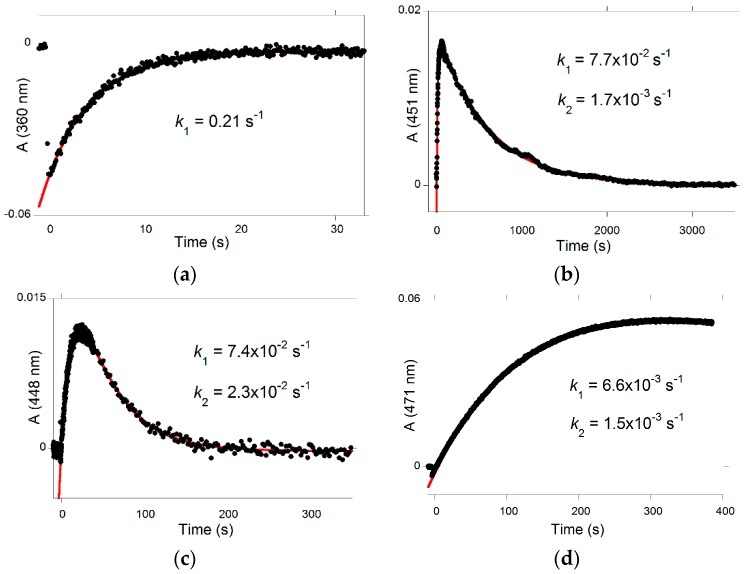
Transient absorption upon a light flash applied to an equilibrated solution of (**a**) P5 at pH = 6.05; (**b**) P4 at pH = 6.06; (**c**) P6 at pH = 5.9; and (**d**) P3 at pH = 6.5.

Glycosidation and/or methylation of DHF also have interesting implications on the photochemical properties of the corresponding pigments. The primary photochemical event is *trans*-*cis* isomerization, and the *cis*-chalcone thus formed decays to products, i.e., the colored flavylium cation or quinoidal base (depending on the pH, forward route) and the colorless *trans*-chalcone (backward route). At near neutral pH, the *cis*-chalcone of P5 (low isomerization barrier) reverts back almost quantitatively to the *trans*-chalcone in a few seconds without forming appreciable amounts of quinoidal base (Figure 13a). By contrast, with P4 (high barrier), the competing backward reaction is much slower, and, after the flash, the flavylium ion accumulates over 1 min, and then the system reverts back to equilibrium in less than 1 h (Figure 13b). As P6 exhibits a lower barrier than P4 (*k*_i_(P6)/*k*_i_(P4) = 17), the visible absorbance is maximized only 25 s after the flash (Figure 13c), and then the system returns to equilibrium in less than 5 min. Finally, P3 and P4 display similar isomerization barriers. However, while irradiation of P4 rapidly forms the flavylium ion, irradiation of P3 more slowly leads to the quinoidal base (cf. the [H^+^]/([H^+^] + *K*_a_) term in Equation (10)). With P3, a maximal accumulation of quinoidal base is observed ca. 5 min after the flash (Figure 13d).

In conclusion, the *O*-glucosides of 4′,7-dihydroxyflavylium and their *O*-methylethers are simple, water-soluble and easily accessible analogs of natural 3-deoxyanthocyanins. This work demonstrates that glycosidation of critical OH groups exerts a subtle influence on the reaction network of flavylium ions and so also on the resulting color and photochromic properties, thus confirming the high versatility of the flavylium multistate.

## 3. Material and Methods

### 3.1. Chemicals

All pigments were chemically synthesized according to already published procedures and completely characterized by NMR and HPLC-DAD-MS analysis [16].

### 3.2. Analyses

Stock solutions of flavylium cations were prepared in 0.1 M HCl. The pH of solutions (measured with a Crison basic 20+ pH meter, Barcelona, Spain) was adjusted by addition of HCl, NaOH and/or pH 8.5 universal buffer (33 mM sodium citrate, 33 mM sodium phosphate, and 57 mM sodium borate). Direct pH jumps were carried out by addition of 1 mL of flavylium cation at pH = 1 to a cuvette (1 cm optical pathlength) containing 1 mL of 0.1 M NaOH and 1 mL of universal buffer previously adjusted at the desired pH with concentrated HCl. Reverse pH jumps were carried out through the addition of HCl to solutions equilibrated or pseudo-equilibrated (i.e., before significant formation of *trans*-chalcone) at slightly acidic or neutral pH values.

UV/Vis absorption spectra were recorded on a Varian Cary 100 Bio or 5000 spectrophotometer (Palo Alto, CA, USA). The stopped-flow experiments were conducted on an Applied Photophysics SX20 stopped-flow spectrometer equipped with a photodiode array detector (Leatherhead, UK). Direct pH jumps investigated by stopped-flow were carried out by charging one syringe (6 mL) with flavylium cation solution at pH = 1 and the other syringe with 4 mL of universal buffer at the desired pH and 2 mL of 0.3 M NaOH. The analyzed solutions were recovered and the pH measured with a glass electrode.

Flash photolysis experiments were performed on a Varian Cary 5000 spectrophotometer with a Harrick fiber-mate (Pleasantville, NY, USA) attached to a Ocean Optics 4-way cuvette holder (Dunedin, FL, USA) to perform light excitation perpendicular to the analyzing beam (sample compartment protected from daylight by black cardboard and black tape). As a pulsed white light source, a commercially available Achiever 630AF camera flash (Hong Kong, China), placed in close contact with the sample holder, was used (time resolution of ca. 0.05 s) [17]. Experiments under continuous irradiation were conducted using a Xenon lamp (excitation band isolated with a monochromator) (Osram, München, Germany). The solutions were irradiated in a quartz cuvette (path length = 1 cm) with magnetic stirring. The absorption spectra were registered before irradiation and at selected time points of irradiation period. The incident light intensity was measured by ferrioxalate actinometry [18].

All curve-fitting procedures were carried out using the solver program from Microsoft Excel 2016 (Redmond, WA, USA).

## Figures and Tables

**Table 1:** Complete sets of thermodynamic and kinetic rate constants characterizing the multistate of the pigments investigated in this work.

**Compound**	**p*K*’_a_**	**p*K*^^^_a_**	**p*K*_a_**	*K*_h_/M *	*K*_t_ *	*K*_i_ *
**DHF**	3.05 ± 0.10	–	4.0 ± 0.1	1.4 × 10^−6^	0.4	1.4 × 10^3^
**P3**	2.30 ± 0.05	4.70 ± 0.05	5.40 ± 0.05	8.9 × 10^−6^	0.60	935
**P4**	2.15 ± 0.05	4.80 ± 0.05	–	8.4 × 10^−6^	1.0	841
**P5**	2.50 ± 0.05	–	3.70 ± 0.05	4.3 × 10^−6^	0.33	2.0 × 10^3^
**P6**	2.60 ± 0.05	5.20 ± 0.05	–	3.6 × 10^−6^	0.68	1.0 × 10^3^
**Compound**	*k*_h_/s^−1^ *	*k*_−h_/M^−1^·s^−1^ *	*k*_t_/s^−1^ *	*k*_−t_/s^−1^ *	*k*_i_/s^−1^ *	*k*_−i_/s^−1^ *
**DHF**	0.02	1.3 × 10^4^	0.22	0.55	0.26	1.8 × 10^−4^
**P3**	0.08	9 × 10^3^	0.36	0.60	5.5 × 10^−3^	5.9 × 10^−6^
**P4**	0.09	1.1 × 10^4^	0.75	0.75	4.7 × 10^−3^	5.6 × 10^−6^
**P5**	0.05	1.3 × 10^4^	0.25	0.75	0.60	3.0 × 10^−4^
**P6**	0.05	1.4 × 10^4^	0.50	0.75	0.08	7.8 × 10^−5^

* Estimated error = 10%; all values obtained at room temperature.

## Supplementary Materials

Supplementary materials can be found at www.mdpi.com/1422-0067/17/10/1751/s1.
