# Supplementary material for: Analogs of Natural 3-Deoxyanthocyanins: O-Glucosides of the 4′,7-Dihydroxyflavylium Ion and the Deep Influence of Glycosidation on Color
Source: Int J Mol Sci. 2016 Oct 20;17(10):1751. doi: 10.3390/ijms17101751 (PMC5085776; doi:10.3390/ijms17101751)
Supplement: Supplementary file 1 [file ijms-17-01751-s001.pdf]

Int. J. Mol. Sci. **2016**, *17*, 1751; doi:10.3390/ijms17101751

Supplementary Materials: Analogs of Natural 3-Deoxyanthocyanins: *O*-Glucosides of the 4′,7-Dihydroxyflavylium Ion and the Deep Influence of Glycosidation on Color

Nuno Basílio, Sheiraz Al Bittar, Nathalie Mora, Olivier Dangles and Fernando Pina

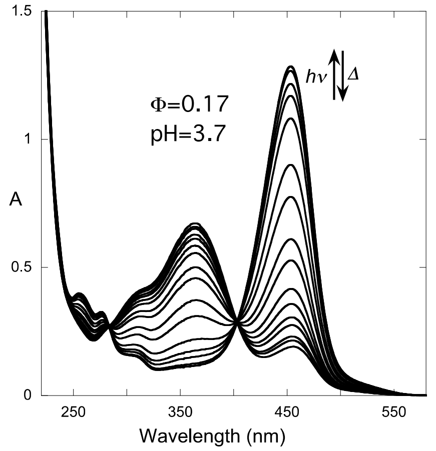

Figure S1. Spectral variations of P3 upon irradiation at 365 nm of an equilibrated solution at pH = 3.7.

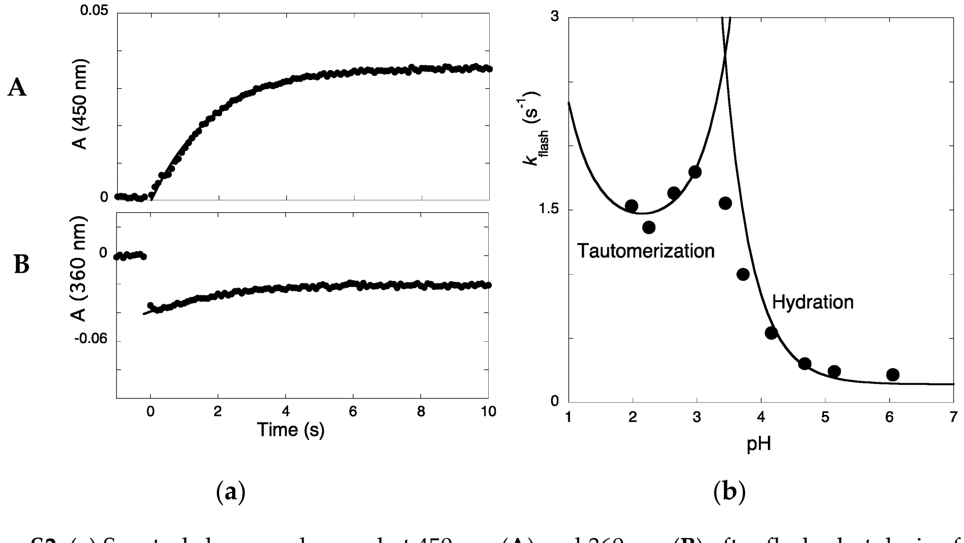

Figure S2. (**a**) Spectral changes observed at 450 nm (**A**) and 360 nm (**B**) after flash photolysis of P5 at pH = 4.2; (**b**) pH-dependence of the apparent first-order rate constants and its fitting according to Equation s (15) and (16): $k_i K_t/(1 + K_t) = 0.15$ s^{-1}; $k_{-h}/(1 + K_t) = 9 \times 10^3$ $M^{-1} \cdot s^{-1}$, $k_h = 0.05$ s^{-1}; $pK_a = 3.7$; $k_i + k_{-t} = 1.35$ s^{-1}; K_a set at $10^{-3.7}$.
